# Supplementary figures and images for: LFRET, a novel rapid assay for anti-tissue transglutaminase antibody detection
Source: PLoS One. 2019 Nov 26;14(11):e0225851. doi: 10.1371/journal.pone.0225851 (PMC6879146; doi:10.1371/journal.pone.0225851)

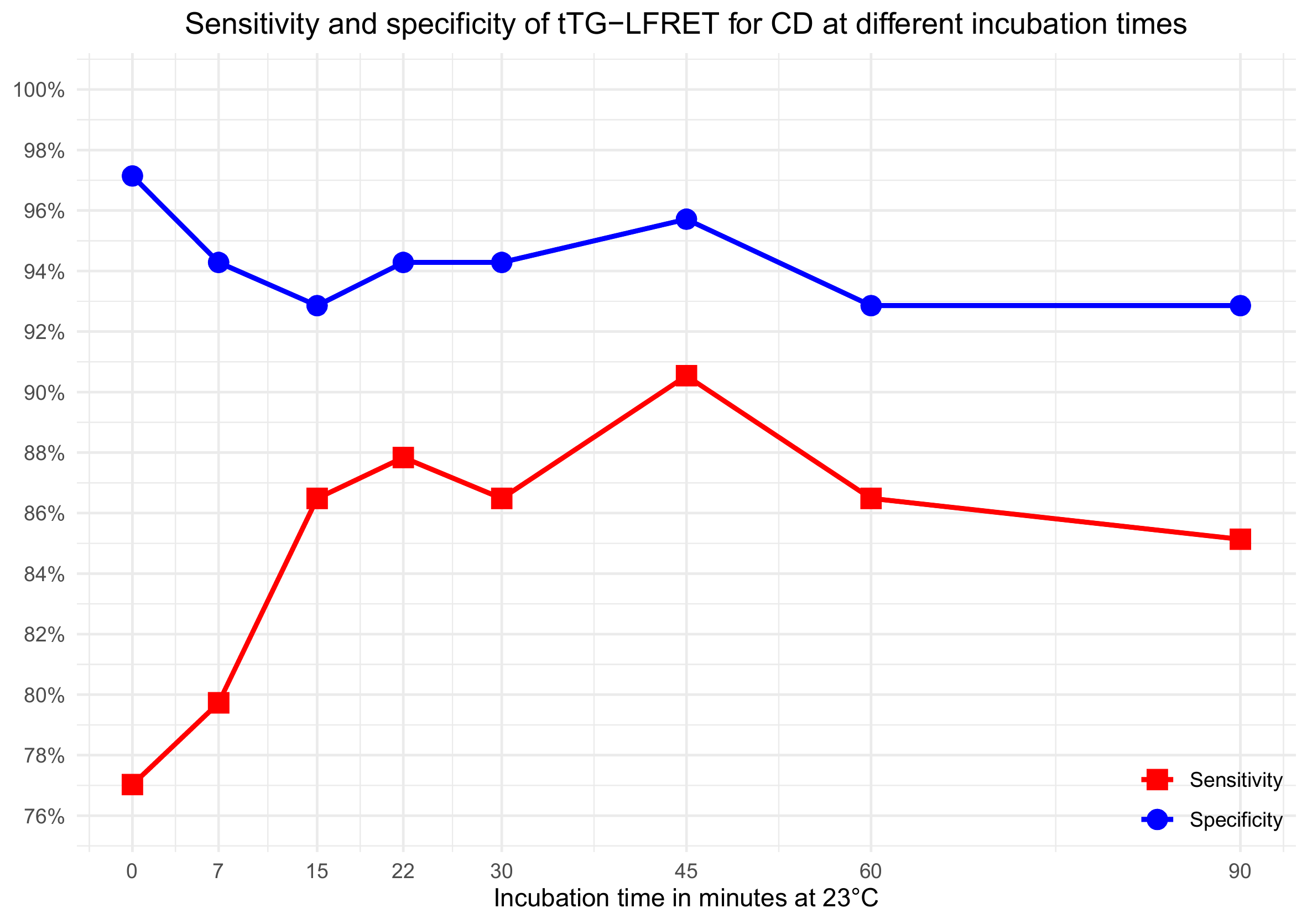

Supplement: S1 Fig — We chose 22 minutes as the incubation time to achieve the best balance between sensitivity, specificity and assay time. (TIF) [file pone.0225851.s001.tif]
