# Supplementary material for: LFRET, a novel rapid assay for anti-tissue transglutaminase antibody detection
Source: PLoS One. 2019 Nov 26;14(11):e0225851. doi: 10.1371/journal.pone.0225851 (PMC6879146; doi:10.1371/journal.pone.0225851)
Supplement: S1 Table — We chose 22 minutes (bolded) as the incubation time to achieve the best balance between sensitivity, specificity and assay time. (DOCX) [file pone.0225851.s002.docx]

| Incubation time (min) | 0 | 7 | 15 | **22** | 30 | 45 | 60 | 90 |
| --- | --- | --- | --- | --- | --- | --- | --- | --- |
| True positive | 57 | 59 | 64 | **65** | 64 | 67 | 64 | 63 |
| False positive | 2 | 4 | 5 | **4** | 4 | 3 | 5 | 5 |
| True negative | 68 | 66 | 65 | **66** | 66 | 67 | 65 | 65 |
| False negative | 17 | 15 | 10 | **9** | 10 | 7 | 10 | 11 |
| Sensitivity | 77,0 % | 79,7 % | 86,5 % | **87,8 %** | 86,5 % | 90,5 % | 86,5 % | 85,1 % |
| Specificity | 97,1 % | 94,3 % | 92,9 % | **94,3 %** | 94,3 % | 95,7 % | 92,9 % | 92,9 % |
